# Extracting Forceps and Key

**Published:** 1844-03

**Authors:** W. W. H. Thackston


### ARTICLE VI.

### *Extracting Forceps and Key.* By W. W. H. THACKSTON, D. D. S.

WE were somewhat surprised to find in a communication from Dr. W. H. Elliot, on the "extraction of teeth," addressed to the "American Journal and Library of Dental Science," the following paragraph: "I cannot agree with Dr. Shepherd that he who has the ability to safely use the key, should not exchange it for any other instrument; nor with Dr. Thackston, that the key should be abandoned entirely. The latter gentleman proves clearly enough that the fulcrum must rest upon the gum in his supposed case, but unfortunately for his argument against the key, he stopped short of telling us how that operation might be performed with the forceps."*

This paragraph refers to an article in reply to a communication on the "turnkey," from my excellent and esteemed friend Dr. Shepherd, written during the period of our studentship; which remark, however, be it distinctly understood, we do not make by way of apology for the doctrine therein maintained; for in a subsequent studentship, and practice (not *quite* as extensive perhaps as Dr. Elliot's) of several years, we have seen nothing which would impel us to a recantation of any single proposition contained in that article; on the contrary, we have experienced the most conclusive evidences of the truth of the principles which we then, in our humble manner, set forth.

In reply to the latter clause of Dr. Elliot's paragraph, as above quoted, we have only to say, that we are at some loss to reconcile the obtuseness of perception, evidenced in the present instance, with the acumen and sagacity displayed by him on other occasions; it is true that we did not, in the article alluded to, state exactly how the force should be applied, or what motions should be given the forceps to effect the evulsion of the teeth, for two very simple and satisfactory reasons; 1st, because the manner of the extraction of the teeth was *not* the point at issue; the subject under discussion being the adaptation and applicability of the instruments usually *employed* in this operation; and 2d, because the presumption with us then *was*, and *now* is, that any one who knew the difference between a turnkey and a pair of forceps, or at least who was at all familiar with the articulation of the teeth, and their anatomical relations, would be at no loss to know how safely to apply the force requisite for their dislodgement, after an effectual hold had been secured; which latter object, I trust, Dr. E. found amply provided for.

We will here, by way of expiating our (in Dr. E's judgment)

* Vide Am. Jour. and Lib. of Dental Science, vol. 4, p. 48.

unfortunate omission, refer him to nearly every work *extant* upon operative dentistry, and especially to Prof. C. A. Harris' and L. Kœcker's instructions on the extraction of teeth with the forceps. All authors since the introduction of improved forceps for this purpose, are, we believe, generally agreed upon the manner of their application; for the removal of the double teeth, the *molares* and *bicuspides*, the requisite motions are an internal, an external lateral, and perpendicular, or as near so as practicable; instead of the lateral motion for the front teeth, the *incisores* and *cuspidati*, they should be rotated, and afterwards removed in the same manner as the *molares,* that is perpendicularly. In this way we may, with the improved forceps, the common, pyramidal screw, the punch, elevator, &c. &c. remove all teeth or fangs, we think, with more ease and facility than with a combination of key and forceps, the key alone, or the lead and rod method, with which Dr. E. seems so much enraptured, but which, to us, appears, we confess, peculiarly objectionable.

In conclusion, we will observe, that we are highly gratified at the inquiring spirit, and disposition to cultivate and improve our science, manifested by Dr. Elliot; such a spirit is praiseworthy and noble, and should be emulated by all; but we will add, that it can never be fostered by illiberality and injustice, and we hope that our friend, Dr. E., will not again inflict that upon us, which he so much complains of in others, viz. unfairness, misrepresentation, &c. &c. We wish the doctor a large accession to his already *extensive* practice, and much success in his scientific investigations.

NOTE.—We are unable, at this moment, to lay our hands upon the article of Dr. Thackston to which Dr. Elliot alludes; but it is our impression that Dr. E's inquiry was, not how the forceps were to be applied in ordinary cases, but to such teeth as presented but one side above the alveolus;—a matter upon which, we confess, we should be glad to be enlightened ourself. M.

